# Supplementary material for: Novel small molecule targeting PgQC reduces Porphyromonas gingivalis virulence
Source: Front Oral Health. 2026 Mar 13;7:1716188. doi: 10.3389/froh.2026.1716188 (PMC13021670; doi:10.3389/froh.2026.1716188)
Supplement: Supplementary file 1 [file Supplementaryfile1.docx]

Inhibition of PgQC by a novel small molecule diminishes pathogenic activity of *Porphyromonas gingivalis*

Nadine Taudte^1^, Linda Liebe^2^, Nadine Jänckel^1^, Daniel Ramsbeck^1,3,4^, Stephan Schilling^3^, Jan Potempa^5,6^, Sigrun Eick^7*^, Mirko Buchholz^1*^

**Supplementary Material**

**Sequence analysis of the N-termini of selected virulence factors of *P. gingivalis* W83**


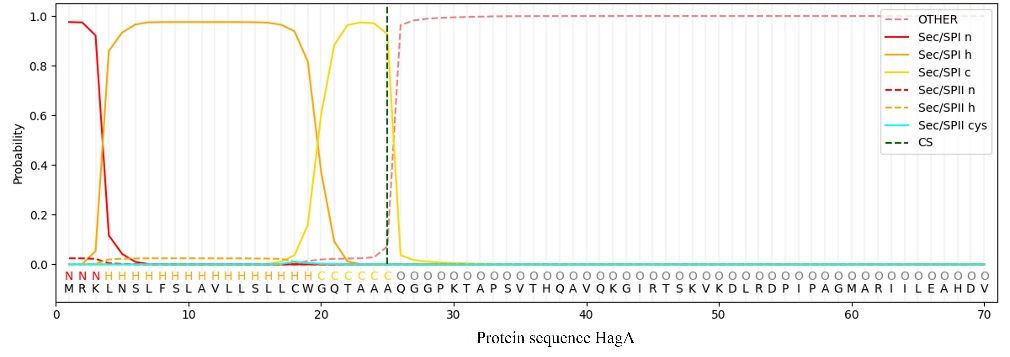


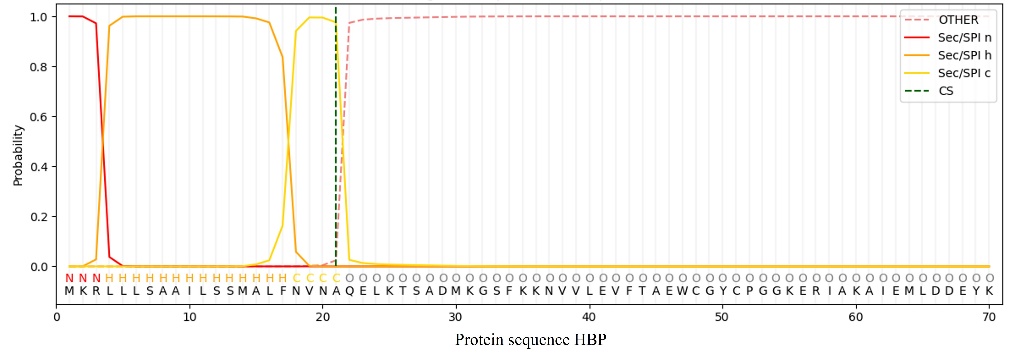


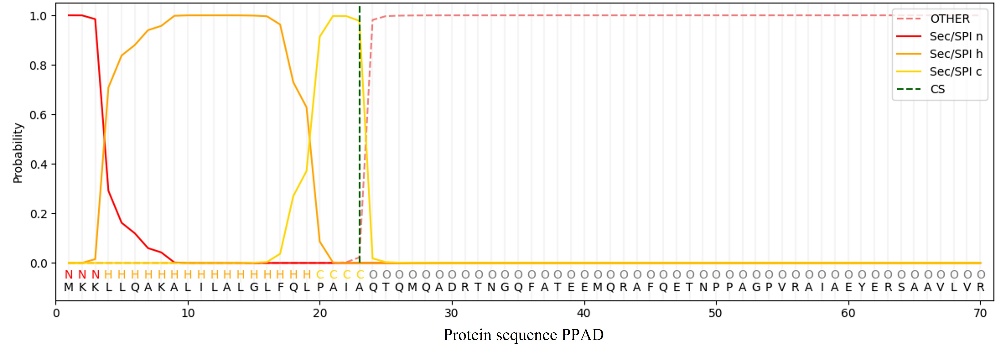


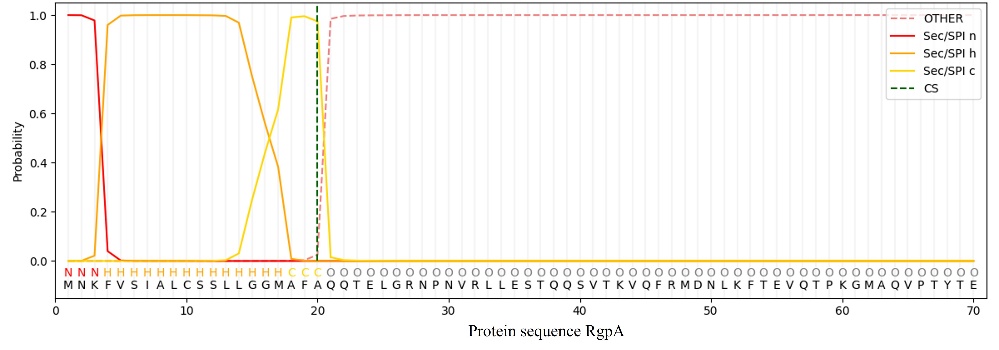


**Supp-Fig 1:** Signal peptide sequences and cleavage site were identified using SignalP ((https://services.healthtech.dtu.dk/services/SignalP-6.0), which enables the classification of N-terminal signal peptides into distinct types, including: Sec/SPI: classical secretory signal peptides, Sec/SPII: lipoprotein signal peptides, Tat/SPI: Tat-dependent signal peptides, Tat/SPII: Tat-lipoprotein signal peptides and Sec/SPIII: pilin-like signal peptides. In all analyzed *P. gingivalis* virulence factors, a conserved glutamine (Q) residue was detected immediately downstream of the predicted cleavage site, suggesting their potential as PgQC substrates.

**Sub-Tab 1: Bacterial strains and culture conditions**

| Strain | Cultivation conditions | Dilution factors in growth experiments |
| --- | --- | --- |
| *Actinomyces oris* | 5% CO_2_, 37°C, TSB-media, Caso Agar | McFarland 0,5, final 1:100 |
| *Rothia dentocariosa* | 5% CO_2_, 37°C, Caso Agar, DSMZ-media 92 | McFarland 0,5, final 1:100 |
| *Corynebacterium matruchotii* | 5% CO_2_, 37°C, Caso Agar, DSMZ- media 92 | McFarland 4, final 1:50 |
| *Streptococcus mitis* | 5% CO_2_, 37°C, Caso Agar, DSMZ- media 92 | McFarland 0.5, final 1:100 |
| *Streptococcus sanguinus* | 5% CO_2_¸37°C, Caso Agar, DSMZ- media 92 | McFarland 0.5, final 1:100 |
| *Neisseria sublava* | 5% CO_2_ ¸37°C, Caso Agar,  TSB media | McFarland 0.5, final 1:100 |
| *Haemophilus parainfluenzae* | 5% CO_2_, 37°C, DSMZ- media 804+Agar-Agar | McFarland 4, final 1:50 |
| *Capnocytophaga granulosa* | anaerob, 37°C, Caso Agar  DSMZ-media 779, | McFarland 4, final 1:50 |
| *Veillonella parvula* | anaerob, 37°C  DSMZ-Medium 136 | McFarland 4, final 1:50 |
| *Lautropia mirabilis* | 37°C, Caso-Agar  DSMZ-Medium 220 | McFarland 4, final 1:50 |

**Synthesis of S-0636**

Scheme 1: Synthesis of Imidazo[4,5-b]pyridine-6-amine

**Synthesis of Imidazo[4,5-b]pyridin-6-amine (5)**

Step 1: **3,5-Dinitropyridin-2-amine (1)**

A solution of 2-chloro-3,5-dinitropyridine (5 g, 24.6 mmol, 1 eq) in ethanol (150 ml) was treated dropwise with concentrated aqueous NH_3_ (7 ml, 123 mmol, 5 eq) a t 0°C. After complete addition, the mixture was stirred at ambient temperature for 1 hour. The solid product was collected by filtration and dried. The compound was used without further purification. Yield: 4.17 g (92%)

Step 2: **5-Nitropyridin-2,3-diamine (2)**

A solution of 3,5-dinitropyridine-2-amine (**1**, 3.9 g, 21.2 mmol, 1 eq) in methanol (80 ml) was treated with 20% aqueous (NH_4_)_2_S (36.1 ml, 106 mmol, 5 eq) and heated to reflux for 1 hour. After cooling, the formed solid was collected by filtration and dried. The product was used without further purification. Yield: 3.2 g (98%); ESI-MS m/z: 155.0 [M+H]^+^; HPLC: rt 4.08 min, >99%; ^1^H-NMR (DMSO-d6) δ: 5.31 (br s, 2H), 6.98 (br s, 2H), 7.36 (d, 1H, ^4^J=2.2 Hz), 8.28 (d, 1H, ^4^J=2.6 Hz)

Step 3: **6-Nitroimidazo[4,5-b]pyridine (3)**

A solution of 5-nitropyridin-2,3-diamine (**2**, 2.7 g, 17.5 mmol) in methanol (14 ml) and triethylorthoformate (14 ml) was stirred at 150°C in a microwave for 10 min. The solvents were evaporated and the residue was used without further purification. Yield: 2.64 g (92%); ESI-MS m/z: 165.1 [M+H]^+^; HPLC: rt 6.21 min, >99%

Step 4: **Imidazo[4,5-b]pyridin-6-amine (4)**

A solution of 6-nitroimidazo[4,5-b]pyridine (**3**, 1240 mg, 7.5 mmol, 1 eq) in aqueous hydrochloric acid (10 %) was treated with SnCl_2_ (5076 mg, 22.5 mmol, 3 eq) and heated in a microwave to 100°C for 30 minutes. After cooling to room temperature, the mixture was basified by means of aqueous NaOH (1 M) and evaporated. The residue was resuspended in MeOH and filtered. The filtrate was evaporated and the reside was purified by flash chromatography (silica, CHCI_3_/MeOH gradient containing 0.5% NH_3_). Yield: 993 mg (98%); ESI-MS m/z: 135.2 [M+H]^+^; HPLC: rt 1.47 min, >99%

Scheme 2: Synthesis of **S-0636** (1-(2-(4-((Imidazo[4,5-b]pyridin-6-ylamino)methyl)-phenoxy)ethyl)guanidine)

**N-(4-(2-Aminoethoxy)benzyl)imidazo[4,5-b]pyridin-6-amine (6)**

Imidazo[4,5-b]pyridin-6-amine (**5**, 268 mg, 2 mmol, 1eq) and N-Boc-(4-(2-aminoethoxy)benzaldehyde (531 mg, 2 mmol, 1eq) were dissolved in EtOH (5ml) and stirred at room temperature for 4 hours. NaBH_4_ (114 mg, 3 mmol, 1.5 eq) was added and stirring was continued overnight. The reaction was quenched by means of water and extracted with EtOAc (3x20 ml). The combined organic layers were washed with brine, dried over Na_2_SO_4_, and evaporated. The residue was purified by flash chromatography (silica, CHCl_3_/MeOH gradient). Boc-deprotection was achieved using TFA/DCM (1:1 (v/v), 2 ml) and TIS (100 µl). After evaporation of the volatiles, the residue was purified by semi-preparative HPLC. Yield: 53 mg (9%); MS m/z: 284.1 [M+H]^+^; HPLC: rt 5.12 min, >99%;
^1^H-NMR (DMSO-d6) δ: 3.18-3.25 (m, 2H); 4.13 (t, 2H, ^3^J=5.0 Hz); 4.31 (s, 2H); 6.94-6.99 (m, 2H); 7.07 (d, 1H, ^4^J=2.4 Hz); 7.33-7.38 (m, 2H); 7.91-8.01 (m, 3H); 8.09 (d, 1H, ^4^J=2.4 Hz); 8.85 (s, 1H).

**1-(2-(4-((Imidazo[4,5-b]pyridin-6-ylamino)methyl)-phenoxy)ethyl)guanidine
(7, S-0636)**

A solution of N-(4-(2-aminoethoxy)benzyl)imidazo[4,5-b]pyridin-6-amine*TFA (**6**, 41 mg, 0.08 mmol, 1 eq) in DMF was treated with N,N'-bis(tert-butoxycarbonyl)-S-methylisothiourea (23 mg, 0.08 mmol, 1 eq), DMAP (1 mg, 0.008 mmol, 0.1 eq) and triethylamine (33 µl, 0.24 mmol, 3 eq). The mixture was stirred for 16 hours at room temperature, quenched with water and extracted with EtOAc (3x20 ml). The combined organic layers were dried over MgCO_3_ and evaporated. Boc deprotection was carried out with TFA/DCM (1:1, 2 ml) and TIS (100 µl). The volatiles were evaporated and the residue was purified by semi-preparative HPLC. Yield: 18 mg (41%); MS m/z: 326.1 [M+H]^+^; HPLC: rt 5.95 min, >99%; ^1^H-NMR (DMSO-d6) δ: 4.04 (t, 2H, ^3^J=5.3 Hz); 4.30 (s, 2H); 6.91-6.95 (m, 2H); 7.07 (d, 1H, ^4^J=2.5 Hz); 7.32-7.36 (m, 2H); 7.64 (t, 1H, ^3^J=5.8 Hz); 8.09 (d, 1H, ^4^J=2.4 Hz); 8.86 (s, 1H).
